# Early Mortality in Cardiac Surgery - Is Lactate Significant?

**DOI:** 10.21470/1678-9741-2023-0245

**Published:** 2024-10-14

**Authors:** Mohammad Zeeshan Hakim, Vivek Tewarson, Sarvesh Kumar, Kumar Rahul, Rati Prabha, Sushil Kumar Singh

**Affiliations:** 1 Department of Cardiovascular and Thoracic Surgery, King George’s Medical University, Lucknow, India; 2 Department of Anaesthesiology, King George’s Medical University, Lucknow, India

**Keywords:** Cardiac Surgical Procedures, Latic Acid, Mortality, Intensive Care Units, Postoperative Period, Length Stay.

## Abstract

**Introduction:**

Serum lactate is a consequence of tissue hypoperfusion and has been used routinely for patient management following cardiac surgery. This study aims to determine the association of lactate with early mortality and postoperative morbidity.

**Methods:**

This is a prospective cohort study carried out in the Department of Cardiovascular and Thoracic Surgery, King George’s Medical University (India), from January 2020 to December 2022. A total of 270 patients were included in this study. Serum lactate levels were measured preoperatively, intraoperatively on-pump, coming off-pump, and at six, 12, 24, and 48 hours postoperatively.

**Results:**

Early mortality was noted in 17 cases (6.3%). While both lactate and lactate clearance correlated with duration of mechanical ventilation, vasopressor duration, and intensive care unit and hospital stay, correlation with early mortality was noted only with lactate at 24 hours. Logistic regression analysis demonstrated that lactate levels at preoperative period (adjusted odds ratio [OR] 4.76 [1.67-13.59], P=0.004) and at 24 hours after bypass (OR 1.21 [1.00-1.47], P=0.046) and vasopressor duration (OR 1.11 [1.04-1.19], P=0.002) are independent predictors of mortality. Receiver operating characteristic curve analysis showed that arterial lactate on-pump, off-pump, and at six, 12, and 24 hours after surgery had significant area under the curve for predicting mortality.

**Conclusion:**

Arterial lactate and lactate clearance show good correlation with duration of mechanical ventilation, vasopressor support, and intensive care unit and hospital stay and can serve as a good indicator to guide therapeutic decisions in postoperative period. However, it fails to be a sensitive predictor of mortality.

## INTRODUCTION

**Table:** 

Abbreviations, Acronyms & Symbols				
ABG	= Arterial blood gas		ICU	= Intensive care unit
AKI	= Acute kidney injury		IQR	= Interquartile range
AF	= Atrial fibrillation		LR	= Likelihood ratio
AUC	= Area under the curve		NPV	= Negative predictive value
BE	= Base excess		NYHA	= New York Heart Association
CPB	= Cardiopulmonary bypass		OR	= Odds ratio
CVP	= Central venous pressure		PPV	= Positive predictive value
ECG	= Electrocardiogram		PRBC	= Packed red blood cell
HbA1C	= Glycated haemoglobin		ROC	= Receiver operating characteristic
IABP	= Intra-aortic balloon pump		SD	= Standard deviation

Since the successful use of cardiopulmonary bypass (CPB) in 1953, several advances in cardiac surgery have made it a less morbid procedure, with a significantly less mortality. There is an increasing interest in the metabolic consequences of CPB, the effects of tissue hypoperfusion, inflammatory responses, use of inotropes, and their association with circulating biomarkers as surrogates of patient morbidity and mortality. Risk stratification using scoring systems such as the European System for Cardiac Operative Risk Evaluation (or EuroSCORE) II or the Society of Thoracic Surgeons (or STS) risk stratification score have been acceptable till date, but being risk assessment tools, they don’t address patient outcome parameters following surgery^[1^,^2]^. Postoperative levels of circulating biomarkers can also serve as prognostic indices of patient outcome in cardiac surgery. Among the various circulating markers, lactate has been studied extensively as a marker of tissue perfusion^[3]^. Arterial lactate level is an important marker for monitoring shock in a wide range of patients and considered a part of the diagnostic criteria for septic shock^[4^-^6]^. Hyperlactatemia is generally considered a serum lactate level > 2 millimoles per litre (mmol/l)^[7]^. The development of hyperlactatemia in patients undergoing surgery on CPB is multifactorial and has been attributed to the time-dependant deleterious effects of CPB on the normal circulatory physiology and cellular function^[8]^. Postoperative lactate levels are being actively researched as markers for adverse outcome and predicting mortality.

This study aims to determine the association between lactate and lactate clearance with early mortality and to determine their significance in postoperative morbidity including duration of vasopressor use, intensive care unit (ICU) stay, and hospital stay.

## METHODS

This is a prospective cohort study carried out in the Department of Cardiovascular and Thoracic Surgery, King George’s Medical University (Lucknow, India), from January 2020 to December 2022. After obtaining ethical clearance from the institute’s ethics committee (103^rd^ ECM II B-Thesis/P14), patients in the age group 18-75 years undergoing open heart surgery on CPB were recruited for the study after taking written informed consent. All procedures were in accordance with the ethical standards of the institutional ethics committee and with the Declaration of Helsinki (1975) and its later amendments or comparable ethical standards. Patients who did not consent for participation and those of age groups < 18 or > 75 years were excluded. Patients undergoing off-pump cardiac procedures, aortic surgery (for dissection/aneurysm), and complex congenital cardiac surgery (including cyanotic congenital heart surgery) and emergency cases were excluded. Patients with uncontrolled diabetes (glycated haemoglobin [HbA1C] > 7), unoptimized thyroid function, chronic kidney disease, chronic liver disease, chronic obstructive pulmonary disease, and those with active Coronavirus disease 2019 infection were also not included in the study.

### Perioperative Management

Intraoperative monitoring included continuous electrocardiogram (ECG), pulse oximetry, invasive blood pressure, central venous pressure (CVP), and urinary output. Induction was done with propofol and fentanyl. Atracurium was utilised as muscle relaxant in all cases. All cases were operated via median sternotomy, and standard surgical technique using CPB with aortic and bi-caval cannulation was employed in all cases. Roller pump CPB, membrane oxygenator, and heparin coated polyvinyl chloride circuits were used. Priming of the CPB circuit was done using 1400 mL of isotonic solution (Stereofundin-Iso, B-braun™), 100 mL of 20% mannitol, and 5000 IU of unfractionated heparin. CPB flow was maintained between 2.2 and 2.5 litres per minute per square metre of body surface area with target mean arterial pressure between 60 and 70 mmHg. Moderate hypothermia (core temperature between 28 and 32 °C) was utilised in all cases and monitored using temperature probe in the oesophagus.

The following definitions were employed.

- Lactate clearance: expressed as a percentage of lactate fall or rise to baseline lactate levels, assessed serially - on-pump, off-pump, and at six, 12, 24, and 48 hours following surgery.
- Vasopressor duration: maximum duration of inotropes (single/multiple) administered to the patient.
- Early mortality: mortality occurring within 30 days following surgery.
- Increase in ICU stay and hospital stay: beyond three-day postoperative ICU stay and five-day postoperative hospital stay.

The patients were monitored postoperatively with regular arterial blood gas (ABG), complete blood counts, liver function test, renal function test, chest X-rays, and continuous ECG. Goal-directed therapy was followed in postoperative period; mean pressures were maintained between 65 and 85 mmHg, systolic pressures < 120 mmHg for cases involving aortic valve replacement, CVP between 8 and 12 cm of water, and urine output between 1 and 1.5 ml/kg/hour. Noradrenaline, adrenaline, and dobutamine were the inotropes used as per the institute protocol. Milrinone was used in patients with right ventricular dysfunction, and vasopressin was used in cases with refractory septic shock. Pre-load and afterload optimisations were done to maintain vitals and CVP. Intra-aortic balloon pump (IABP) was used when indicated. Blood products were transfused to maintain haemoglobin levels ≥ 9 gm/dl.

Arterial lactate was derived from ABG sampling performed during induction of anaesthesia preoperatively, intraoperatively on-pump (immediately after going on CPB), after coming off-pump (following cessation of CPB support), and at six, 12, 24, and 48 hours postoperatively (using GEM Premier 3000, Blood/Gas Electrolyte analysers; Instrumentation Laboratory, Le Pré-Saint-Gervais, France). Lactate clearance was calculated at the aforementioned intervals. Duration of vasopressor use, hospital and ICU stays, and early mortality (defined as occurring within 30 days of surgery) were recorded.

Continuous variables are expressed as mean ± standard deviation or median [interquartile range], discrete variables are expressed as proportions. Continuous variables were assessed using independent *t*-test or Mann-Whitney U test, whichever was applicable. Discrete variables were assessed using Fischer’s exact test or Chi-square test as applicable. Pearson’s correlation analysis was done for correlating the continuous outcome variables and the assessed biomarkers. Logistic regression using “forward LR” model was used to identify independent predictors of mortality. Variables that were significant in the univariate analysis (*P*<0.05) were included in the logistic regression model. Survival analysis was carried out to plot receiver operating characteristic (ROC) curve and to determine area under the curve (AUC) in order to determine the sensitivity, specificity, and cutoff values for the studied markers. *P*-value of < 0.05 has been considered statistically significant. Data analysis was done using SPSS software version 16.0 (SPSS Inc, Chicago, Illinois).

## RESULTS

**Fig. 1:**
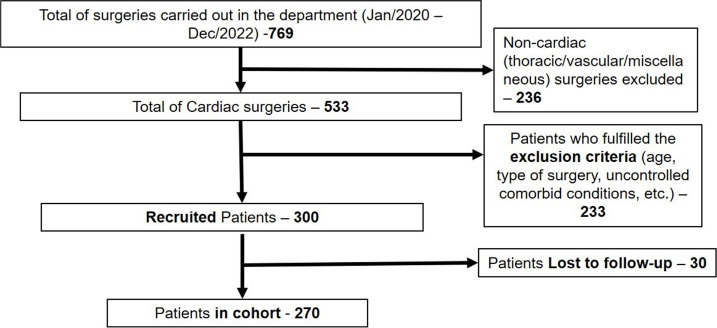
Research methodology - patient selection process.

As depicted in Figure 1, 300 patients who fulfilled the inclusion criteria were initially recruited. Thirty patients were lost to follow-up and were further excluded from the study. Therefore, a total of 270 patients were included in this study. The demographic and clinical laboratory findings are detailed in Table 1. The mean age was 38.1 ± 14.2 years, with most patients belonging to < 50-year age group, and there was roughly equal gender distribution. Of the patients, 7.4% were smokers, and 4.8% were diabetic; 83% presented with New York Heart Association (NYHA) class III, and the median preoperative ejection fraction was 58% [54-62]; 74.8% cases had valvular pathology, while the rest had coronary artery disease, congenital heart disease, and tumours. The median bypass time in the study group was 71 [55-103.5] minutes; and median cross-clamping time was 34 [25-62] minutes. Of all the cases, 7.4% needed intraoperative defibrillation, while 2.6% required IABP support. The median duration of mechanical ventilatory support in the postoperative period was 6.5 hours^[6^-^9]^, while a median of 1 unit of packed red cell (PRC) was transfused in the patients^[1^-^2]^. The average length of ICU stay was 3.08 ± 0.86 days, while the average postoperative hospital stay was 5.25 ± 1.98 days. The median lactate levels showed a steady rise till the end of the first postoperative day and resolved by 48 hours following surgery. We observed early mortality in 17 cases (6.3%). While low cardiac output syndrome and right ventricular failure were the leading causes, infection resulting in ventilator-associated pneumonia and sepsis were also notable.

**Table 1:** Patients’ demographic profile and clinical, procedural, and laboratory parameters.

Variables	n (270)
Age (mean ± SD, years)	38.1 ± 14.2
Males (n, %)	139 (51.48)
Comorbidities (n, %)	
Diabetes mellitus	13 (4.8)
Hypertension	2 (0.7)
Hypothyroidism	7 (2.6)
Personal history (n, %)	
Alcohol and smoking	5 (1.9)
Smoking	20 (7.4)
None	245 (90.7)
Preoperative NYHA (n, %)	
I	0 (0)
II	41 (15.2)
III	224 (83)
IV	5 (1.9)
Preoperative ejection fraction (median [IQR], %)	58 [54-62]
Type of surgery (n, %)	
Valve replacement	202 (74.8)
Coronary artery bypass grafting	31 (11.4)
Tumour excision	4 (1.5)
Congenital cardiac lesions (adult)	33 (12.2)
Bypass time (median [IQR], minutes)	71 [55-103.5]
Cross-clamping time (median [IQR], minutes)	34 [25-62]
Intraoperative defibrillation (n, %)	20 (7.4)
Use of IABP in postoperative period (n, %)	7 (2.6)
Vasopressor duration (median [IQR], hours)	4.5 [2-9]
Duration of mechanical ventilatory support (median [IQR], hours)	6.5 [6-9]
Preoperative haemoglobin (mean ± SD, gram/decilitre)	12.94±1.77
Perioperative PRBC transfusions (median [IQR], units transfused)	1 [1-2]
Length of ICU stay (mean ± SD, days)	3.08 ± 0.86
Length of postoperative hospital stay (mean ± SD, days)	5.25 ± 1.98
Mortality (n, %)	17 (6.3%)
Right ventricular failure	6 (2.22)
Low cardiac output syndrome	5 (1.85)
Ventilator-associated pneumonia	3 (1.11)
Sepsis	3 (1.11)
Arterial lactate (median [IQR], millimole per litre)	
Preoperative	1.4 [1.2-1.9]
On-pump (commencement of CPB)	2.9 [2.4-3.8]
Off-pump (at termination of CPB)	3.5 [2.8-4.4]
6 hours following surgery	3.8 [2.9-4.9]
12 hours following surgery	2.9 [2.2-3.9]
24 hours following surgery	2.1 [1.5-2.9]
48 hours following surgery	1.4 [1.1-1.8]
Lactate clearance (%)	
On-pump (commencement of CPB)	-95.6 [-157.3 to -47.4]
Off-pump (termination of CPB)	-137.5 [-212.9 to -74.4]
6 hours following surgery	-142.9 [-238.8 to -85.9]
12 hours following surgery	-92.9 [-175 to -42.9]
24 hours following surgery	-44.4 [-104.2 to 0.00]
48 hours following surgery	0.00 [-33.3 to 28.6]

CPB=cardiopulmonary bypass; IABP=intra-aortic balloon pump; ICU=intensive care unit; IQR=interquartile range; NYHA=New York Heart Association; PRBC=packed red blood cell; SD=standard deviation

Univariate analysis (Table 2) demonstrated a significantly higher early mortality in patients with diabetes, intraoperative use of defibrillation, and IABP use. The early mortality group had a prolonged duration of ventilation and inotrope use, whereas postoperative ICU and hospital stay were shorter. Serial lactate levels significantly raised from the preoperative period to 24 hours following surgery in the mortality group, however, lactate clearance was not significantly different (Table 3).

**Table 2:** Association of demographic profile and clinical parameters and lactate with mortality.

Variables	Survivors (n=253)	Early mortality (n=17)	*P*-value
Age (mean ± SD, years)	37.7 ± 13.8	43.76 ± 18.9	0.197
Males (n, %)	127 (50.2)	8 (47.1)	1.00
Comorbidities (n, %)			0.031
Diabetes mellitus	10 (4)	3 (17.6)	
Hypertension	1 (0.4)	1 (5.9)	
Hypothyroidism	7 (2.8)	0 (0)	
Epilepsy	1 (0.4)	0 (0)	
Personal history (n, %)			1.00
Alcohol and smoking	5 (2)	0 (0)	
Smoking	19 (7.5)	1 (5.9)	
None	229 (90.5)	16 (94.1)	
Preoperative NYHA (n, %)			0.059
I	0 (0)	0 (0)	
II	36 (14.2)	5 (29.4)	
III	213 (84.2)	11 (64.7)	
IV	4 (1.6)	1 (1.9)	
Preoperative ejection fraction (median [IQR], %)	58 [54-62]	56 [53-59.5]	0.262
Type of surgery (n, %)			0.910
Valve replacement	191 (75.5)	11 (64.7)	
Coronary artery bypass grafting	28 (11.0)	4 (23.5)	
Tumour excision	3 (1.2)	0 (0)	
Congenital cardiac lesions (adult)	31 (12.3)	2 (11.8)	
Bypass time (median [IQR], hours)	70 [54-103.5]	82 [58.8-140.2]	0.254
Cross-clamping time (median [IQR], hours)	34 [25-63]	30 [23-47.5]	0.201
Intraoperative defibrillation (n, %)	16 (6.3)	4 (23.5)	0.028
Use of IABP in postoperative period (n, %)	2 (0.8)	5 (29.4)	< 0.001
Vasopressor duration (median [IQR], hours)	4 [2-8]	12 [4.5-34]	< 0.001
Duration of mechanical ventilatory support (median [IQR], hours)	6.5 [6-9]	11 [6-37]	0.039
Preoperative haemoglobin (mean ± SD, gram/ decilitre)	13.98 ± 3.02	12.88 ± 1.65	0.920
PRBC transfusions (median [IQR], units transfused)	1 [1-2]	1 [1-2]	0.058
Length of ICU stay (mean ± SD, days)	3 [3-3]	2 [1-3]	< 0.001
Length of postoperative hospital stay (median [IQR], days)	5 [5-5]	2 [1-3]	< 0.001

IABP=intra-aortic balloon pump; ICU=intensive care unit; IQR=interquartile range; NYHA=New York Heart Association; PRBC=packed red blood cell; SD=standard deviation

**Table 3:** Association of lactate values with early mortality.

	Survivors (n=253)	Early mortality (n=17)	*P*-value
Arterial lactate (median [inter-quartile range], millimole per litre)			
Preoperative			
On-pump (commencement of CPB)	1.4 [1.2-1.9]	1.9 [1.5-2.9]	0.017
Off-pump (at termination of CPB)	2.9 [2.4-3.7]	4.3 [2.9-5.2]	0.002
6 hours following surgery	3.5 [2.7-4.3]	4.7 [3.2-7.9]	0.003
12 hours following surgery	3.7 [2.8-4.8]	6.2 [3.9-10]	< 0.001
24 hours following surgery	2.8 [2.1-3.8]	10.2 [2.6-13.9]	0.002
48 hours following surgery	2.1 [1.5-2.9]	9.4 [1.5-14.6]	0.031
	1.4 [1.1-1.8]	3.3 [1.1-13.8]	0.063
Lactate clearance (%)			
On-pump (commencement of CPB)	-96 [-157.5 to -47.5]	-71.4 [-176.3 to -24.3]	0.698
Off-pump (termination of CPB)	-138.5 [-210 to -76.2]	-100 [-348.9 to -8.9]	0.745
6 hours following surgery	-142.9 [-237.9 to -91.9]	-131.9 [-543.6 to -34.4]	0.846
12 hours following surgery	-92.4 [-169 to -43.1]	-158.6 [-837.5 to 27.6]	0.224
24 hours following surgery	-44.4 [-100 to 0.00]	-134.6 [-715.2 to 16.9]	0.367
48 hours following surgery	0.00 [-31.3 to 28.6]	-13.8 [-396.3 to 36.8]	0.343

CPB=cardiopulmonary bypass

Duration of mechanical ventilatory support and vasopressor duration showed significant correlation with lactate and lactate clearance from to six to 48 hours following surgery. Correlation of ICU stay was significant with lactate in preoperative period, while on-pump, and at 24 and 48 hours following surgery, while lactate clearance correlated significantly with ICU stay from six through 48 hours. Postoperative hospital stay correlated only at preoperative lactate levels and lactate clearance at 48 hours (Table 4).

**Table 4:** Correlation of lactate and lactate clearance with duration of inotrope requirement, mechanical ventilatory support, ICU stay, and hospital stay.

Inotropic support duration	Pearson’s correlation coefficient	*P*-value
Arterial lactate		
Preoperative	-0.002	0.976
On-pump (commencement of CPB)	0.031	0.611
Off-pump (at termination of CPB)	0.068	0.265
6 hours following surgery	0.250	< 0.001
12 hours following surgery	0.344	< 0.001
24 hours following surgery	0.388	< 0.001
48 hours following surgery	0.412	< 0.001
Lactate clearance (%)		
On-pump (commencement of CPB)	-0.086	0.158
Off-pump (termination of CPB)	0.110	0.071
6 hours following surgery	-0.288	< 0.001
12 hours following surgery	-0.375	< 0.001
24 hours following surgery	-0.408	< 0.001
48 hours following surgery	-0.533	< 0.001
Duration of mechanical ventilation	Pearson’s correlation coefficient	*P*-value
Arterial lactate		
Preoperative	0.006	0.924
On-pump (commencement of CPB)	0.016	0.788
Off-pump (at termination of CPB)	0.124	0.042
6 hours following surgery	0.198	0.001
12 hours following surgery	0.280	< 0.001
24 hours following surgery	0.340	< 0.001
48 hours following surgery	0.407	< 0.001
Lactate clearance (%)		
On-pump (commencement of CPB)	0.004	0.948
Off-pump (termination of CPB)	-0.072	0.238
6 hours following surgery	-0.121	0.048
12 hours following surgery	-0.185	0.003
24 hours following surgery	-0.217	< 0.001
48 hours following surgery	-0.318	< 0.001
ICU stay	Pearson’s correlation coefficient	*P*-value
Arterial lactate		
Preoperative	-0.196	0.001
On-pump (commencement of CPB)	-0.167	0.006
Off-pump (at termination of CPB)	-0.08	0.186
6 hours following surgery	0.023	0.703
12 hours following surgery	0.088	0.153
24 hours following surgery	0.160	0.009
48 hours following surgery	0.309	< 0.001
Lactate clearance		
On-pump (commencement of CPB)	-0.006	0.928
Off-pump (termination of CPB)	-0.03	0.624
6 hours following surgery	-0.187	0.002
12 hours following surgery	-0.255	< 0.001
24 hours following surgery	-0.317	< 0.001
48 hours following surgery	-0.617	< 0.001
Postoperative hospital stay	Pearson’s correlation coefficient	*P*-value
Arterial lactate		
Preoperative	-0.164	0.007
On-pump (commencement of CPB)	-0.122	0.045
Off-pump (at termination of CPB)	-0.068	0.265
6 hours following surgery	-0.108	0.077
12 hours following surgery	-0.123	0.045
24 hours following surgery	-0.101	0.100
48 hours following surgery	0.003	0.961
Lactate clearance (%)		
On-pump (commencement of CPB)	-0.005	0.934
Off-pump (termination of CPB)	0.01	0.868
6 hours following surgery	-0.026	0.674
12 hours following surgery	-0.01	0.872
24 hours following surgery	-0.026	0.667
48 hours following surgery	-0.198	0.001

CPB=cardiopulmonary bypass; ICU=intensive care unit

Our logistic regression analysis included the variables that had been significant in the univariate analysis, *i.e.*, diabetes, use of IABP, intraoperative defibrillation, vasopressor duration, ventilatory support duration, packed red cells transfused, length of ICU stay, and length of hospital stay, along with lactate values from the preoperative period to 24 hours following surgery. The logistic regression analysis demonstrated lactate levels at preoperative period (adjusted odds ratio [OR] 4.76 [1.67-13.59], *P*=0.004), at 24 hours after bypass (OR 1.21 [1.00-1.47], *P*=0.046), and vasopressor duration (OR 1.11 [1.04-1.19], *P*=0.002) as key independent predictors of mortality (Table 5).

**Table 5:** Logistic regression analysis depicting independent predictors of in-hospital mortality.

Variables	*P*-value	Adjusted odds ratio
Preoperative lactate	0.004	4.76 [1.67-13.59]
Lactate 24 hours following surgery	0.046	1.21 [1.00-1.47]
Vasopressor duration	0.002	1.11 [1.04-1.19]

**Fig. 2:**
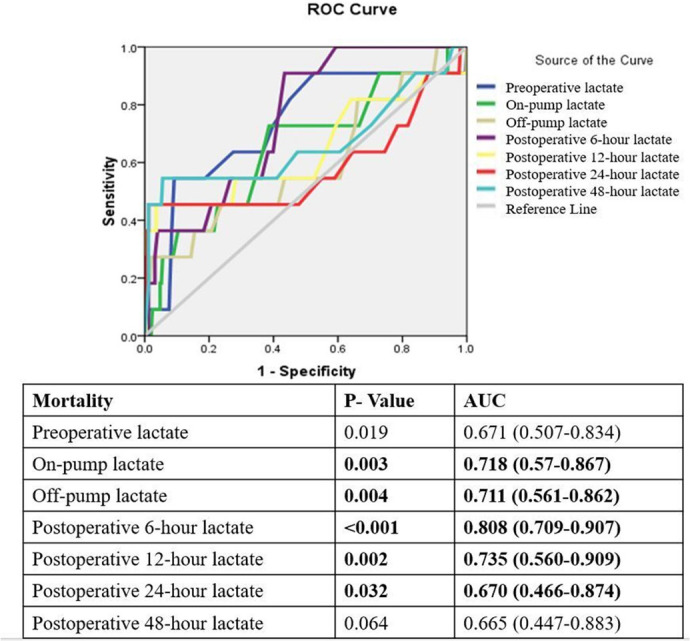
Receiver operating characteristic (ROC) curve analysis for lactate at various intervals and mortality. AUC=area under the curve.

In the ROC curve analysis (Figure 2), on-pump, off-pump, and at six, 12, and 24 hours following surgery arterial lactate had significant AUC for mortality.

Arterial lactate ≥ 7.3 at 24 hours had the highest specificity (98.1%) for predicting mortality, but with a poor sensitivity (57.1%). The highest sensitivity of arterial lactate for mortality was 76.5% for on-pump arterial lactate levels ≥ 3.25 mmol/l (Table 6).

**Table 6:** Sensitivity, specificity, positive predictive value, negative predictive value, and likelihood ratios of lactate at the preoperative period, intraoperatively on-pump, after coming off-pump, and at six, 12, 24, and 48 hours postoperatively.

Lactate	Cutoff	Sensitivity	Specificity	PPV	NPV	LR+	LR-
Preoperative lactate	≥ 1.55	70.6	59.7	10.5	96.7	1.75	0.49
On-pump	≥ 3.25	76.5	61.3	11.7	97.4	1.98	0.38
Off-pump	≥ 3.65	70.6	56.1	9.8	96.6	1.61	0.52
6-h lactate	≥ 4.05	75	61.5	11.6	97.3	1.95	0.41
12-h lactate	≥ 3.65	66.7	71.4	13.6	96.9	2.33	0.47
24-h lactate	≥ 7.3	57.1	98.1	66.8	97.1	30.1	0.43
48-h lactate	≥ 3.25	54.5	94.4	35.54	96.27	8.20	0.58

LR=likelihood ratio (positive and negative); NPV=negative predictive value; PPV=positive predictive value

## DISCUSSION

In our cohort of 270 patients, the early mortality group had a prolonged duration of ventilation, inotrope use, and postoperative ICU and hospital stays. We also found that patients with diabetes and intraoperative use of defibrillation had a significantly higher mortality. While our patients had optimised blood sugars and HbA1C was < 8 in all cases, the overall effects of hyperglycaemia and response to surgical stress may be important factors that affect the outcome. Diabetes - especially uncontrolled - can lead to increased morbidity, however, optimum preoperative level of HbA1C is still being debated. Diabetes itself has been linked to increased incidence of prolonged ICU stay, sternal instability and/or dehiscence, respiratory insufficiency, delirium, stroke, renal dysfunction, and postoperative reintubation^[9^,^10]^. The use of defibrillation was higher in the mortality subgroup, corroborating with data from few studies which also found increased in-hospital mortality for patients receiving treatment for perioperative ventricular fibrillation. This can be explained by the fact that while coming off pump, ventricular fibrillation results in subendocardial ischemia that, in turn, results in myocardial dysfunction^[11^,^12]^.

We also found that lactate levels were significantly elevated in the mortality group from the preoperative period till 24 hours following surgery, however, lactate clearance did not show significant difference between the two groups. Lactate and lactate clearance both correlated significantly with duration of mechanical ventilatory support and vasopressor duration in the postoperative period. ICU stay and hospital stay also correlated with the lactate and lactate clearance levels. This indicates that elevated lactate levels have a potential impact on patients undergoing on-pump cardiac surgery.

Hyperlactatemia in cardiac surgery has been linked to prolonged CPB time, which is attributed to tissue hypoperfusion that, in turn, is a result of CPB use^[8^,^13]^. However, age, complexity and urgency of surgery, blood transfusion, pH during CPB, status of venous return, use of lactate containing priming solutions, diabetes, use of milrinone, propofol and norepinephrine, and renal function have been noted as significant causes of postoperative hyperlactatemia. A few important outcome parameters affected by this ensuing hyperlactatemia during CPB include prolonged ICU stay, duration of mechanical ventilatory support, hospital stay, risk of developing acute renal failure, respiratory distress, pneumonia, and circulatory disorders. The arterial lactate level itself has also been investigated, suggesting that normal lactate levels during CPB may well exceed the normal limits^[8^,^14^,^15]^.

The question as to how much lactate levels can be considered as significant, when these levels should be evaluated, and how they reflect on the patient outcome is a matter of ongoing research.

Kogan et al.^[16]^ had demonstrated a significant association between arterial lactate levels > 4.4 mmol/l during the first postoperative 10 hours with prolonged ventilation time, longer ICU stay, and increased mortality. Algarni et al.^[17]^, in a study on 307 cardiac surgery patients, had found significant association between early hyperlactatemia (lactate > 3 mmol/l, within 24 hours of surgery) and increased CPB time, requirement of postoperative extracorporeal membrane oxygenation support (0% *vs.* 5.7%, *P*<0.0001), increased hospital mortality, and prolonged ICU stay.

In a retrospective analysis of 7,447 patients, Duval et al.^[18]^ found that while the median ∆-lactate of most patients undergoing cardiac surgery was 0.6 (0.3-1) mmol/L (∆-lactate was defined as the difference between the highest intraoperative blood lactate and the baseline lactate level), most of patients (65.9%) exhibited a ∆-lactate between 0.1 and 0.9 mmol/L. There was a concentration-dependent relationship between ∆-lactate and 30-day mortality (as of a 1 mmol/L increase). Haanschoten et al.^[19]^ had determined that postoperative peak arterial levels can serve as independent predictors for 30-day mortality (OR 1.44 [1.39-1.48], *P*<0.001) as well as for late mortality (hazard ratio 1.05 [1.01-1.10], *P*<0.025). Patra et al.^[20]^ (2019), in their retrospective analysis of 362 open heart surgery patients, had stated that lactate levels at ICU admission and 12-hour blood lactate level were significant predictors of complications. Whereas, 24-hour blood lactate level was significantly associated with prolonged ICU stay and hospital stay.

Matteucci et al.^[21]^, in their study of 1,099 adult patients undergoing on-pump surgery, noted that 33.8% of patients developed hyperlactatemia (lactate > 2 mmol/l) postoperatively. Emergency procedure, CPB duration, and aortic cross-clamping time were independently associated with hyperlactatemia. These patients had significantly prolonged ventilation time, hospital stay, vasopressor requirement, IABP support, and 30-day mortality. Kaplan-Meier curve showed worse long-term survival (mean follow-up: 4.02 ± 1.58 years) in these patients.

Naik et al.^[8]^, in their retrospective analysis of 370 patients undergoing cardiac surgery on CPB, found that there was a significant correlation between aortic cross-clamping time and CPB time with peak intraoperative blood lactate levels. Patients with hyperlactatemia had significantly higher rate of postoperative morbidity like prolonged requirement of inotropes, atrial fibrillation (AF), and prolonged ICU and hospital stays.

To further understand the utility of both lactate and lactate clearance as predictors of mortality, we used multivariate logistic regression analysis, which suggested that preoperative lactate, lactate at 24 hours, and vasopressor duration were independent predictors of early mortality in this cohort. Furthermore, the adjusted odds for mortality were highest for preoperative lactate levels (OR 4.76 [1.67-13.59], *P*=0.004). Given the abovementioned findings, we can conclude that despite being correlated with duration of ventilatory and vasopressor supports, lactate clearance does not have much significant impact on predicting mortality.

In the ROC curve analysis, we found that on-pump lactate levels > 3.25 mmol/l had the highest sensitivity (76.5%), with a likelihood ratio of 1.98 in predicting mortality. Lactate levels > 7.3 at 24 hours following CPB had maximum specificity (98.1%). We can infer that although very specific at 24 hours, lactate levels don’t provide high sensitivity for use in predictive models for risk stratification.

Kim et al.^[22]^ (2020), in a study involving 207 adult congenital heart surgery patients, had found that lactate was elevated > 5 mmol/L in 42% of the cases. They could not find any significant difference among different lactate level groups in hospital stay, ICU stay, ventilation requirement, acute kidney injury (AKI), need for redo surgery, or rates of hospital or ICU readmission. In multivariable analysis, lactate levels were not a significant predictor of either hospital length of stay or AKI.

Considering the complexity of biochemical responses and alteration in physiology, perhaps using lactate as a stand-alone marker for predicting mortality may not yield better results. In a study including 1,058 patients and comparing base excess (BE) and lactate for determining ICU mortality after cardiac surgery, Zante et al.^[23]^ found that lactate levels > 3.9 mmol/l at ICU admission had 61.9% sensitivity and 87.5% specificity for predicting mortality. Their subgroup analyses revealed a combination of lactate ≤ 3.9 mmol/l and BE ≥ -6.7 had stronger impact on mortality than a combination of lactate of > 3.9 mmol/l and BE > -6.7 (hazard ratio 2.56, 95% confidence interval 0.18-37.17). This can indicate that BE might be superior to lactate in predicting ICU mortality after cardiac surgery.

The level of lactate in patients undergoing surgery on CPB is also a matter of debate and despite authors recommending higher than normal lactate values as cutoffs, our findings suggest that on-pump lactate of > 3.25 mmol/l is most sensitive. Svenmarker et al.^[14]^ had also deduced an on-pump lactate of > 2 mmol/l as a significant predictor for mortality. Therefore, it can benefit the patients if on-pump lactates are kept within range to avoid postoperative complications.

### Limitations

Although all attempts have been made to rule out bias, we can narrow down a few limitations to our study. One of these limitations is the exclusion of emergency cases. Although the purpose of the study is to assess the role of lactate in predicting mortality, it is understood that patients undergoing emergency surgery have a grave prognosis, especially when they are already in failure. This was the rationale for excluding emergency cases, in order to create a uniform cohort for the assessment of lactate. However, further insight is needed in this matter. Similarly, the exclusion of complex congenital cases (especially cyanotic heart diseases) and aortic surgeries has been done as this subset of patients will behave differently due to an increased degree of complexity involved in the procedure, that increases the CPB duration, degree of tissue ischemia, as well as the need for postoperative life support strategies, including mechanical circulatory supports. These confounding factors could alter the outcome to our hypothesis - is lactate a sensitive marker in predicting early mortality? Also, our findings are based on utilising total vasopressor duration, that may not be as sensitive as vasoactive inotropic score for predicting mortality following cardiac surgery^[24]^. However, as the study was aimed at evaluating lactate as a predictor of mortality, we utilised the vasopressor duration as a representation for inotropic requirements.

## CONCLUSION

We can conclude that arterial lactate and lactate clearance show good correlation with duration of mechanical ventilation, vasopressor support, ICU stay, and hospital stay, and can serve as a good indicator to guide therapeutic decisions in the postoperative period, however, it failed to serve as a sensitive marker (maximum sensitivity on-pump of 76.5% for arterial lactate > 3.25 mmol/l) to predict mortality. Therefore, further attention needs to be focussed on other markers such as BE, lactate clearance, and ∆-lactate to assess their utility in predicting mortality.
